# Mechanism of Arctigenin-Induced Specific Cytotoxicity against Human Hepatocellular Carcinoma Cell Lines: Hep G2 and SMMC7721

**DOI:** 10.1371/journal.pone.0125727

**Published:** 2015-05-01

**Authors:** Zheng Lu, Shengbo Cao, Hongbo Zhou, Ling Hua, Shishuo Zhang, Jiyue Cao

**Affiliations:** 1 College of Veterinary Medicine, Huazhong Agricultural University, Wuhan, China; 2 Department of Veterinary Medicine, Rongchang Campus of Southwest University, Chongqing, China; University of Quebec at Trois-Rivieres, CANADA

## Abstract

Arctigenin (ARG) has been previously reported to exert high biological activities including anti-inflammatory, antiviral and anticancer. In this study, the anti-tumor mechanism of ARG towards human hepatocellular carcinoma (HCC) was firstly investigated. We demonstrated that ARG could induce apoptosis in Hep G2 and SMMC7721 cells but not in normal hepatic cells, and its apoptotic effect on Hep G2 was stronger than that on SMMC7721. Furthermore, the following study showed that ARG treatment led to a loss in the mitochondrial out membrane potential, up-regulation of Bax, down-regulation of Bcl-2, a release of cytochrome c, caspase-9 and caspase-3 activation and a cleavage of poly (ADP-ribose) polymerase in both Hep G2 and SMMC7721 cells, suggesting ARG-induced apoptosis was associated with the mitochondria mediated pathway. Moreover, the activation of caspase-8 and the increased expression levels of Fas/FasL and TNF-α revealed that the Fas/FasL-related pathway was also involved in this process. Additionally, ARG induced apoptosis was accompanied by a deactivation of PI3K/p-Akt pathway, an accumulation of p53 protein and an inhibition of NF-κB nuclear translocation especially in Hep G2 cells, which might be the reason that Hep G2 was more sensitive than SMMC7721 cells to ARG treatment.

## Introduction

Liver cancer (LC) ranks the fifth among common cancers in the world, which results in poor prognosis and no effective systematic treatment options available for patients at present. Unfortunately, it exhibits an annually increasing occurrence worldwide and mainly in developing countries [1,2]. According to reports, various risk factors could result in hepatocarcinogenesis, including excessive alcohol drinking, chronic hepatitis B virus (HBV) or hepatitis C virus (HCV) infections, cirrhosis, carcinogen exposure (such as aflatoxin B1), and a number of genetic and epigenetic alterations [3,4]. Since LC is characterized as high chemoresistance, liver transplantation or surgical resection may offer an early cure in LC, but still be ineffective to the majority of patients (>80%) with unresectable advanced disease. Hence, this emphasizes the need for seeking novel compounds with higher activity and less side effects for chemoprevention and treatment.

Apoptosis, a type of programmed cell death, is not only a fundamental cellular event during development, but also a critical mechanism of cell death in response to anti-cancer drugs in the therapy of cancer [5]. Most cancer therapeutic approaches including radiation and chemotherapy inhibit tumor growth via inducing cancer cells apoptosis [6]. It has been well established that apoptosis is mainly mediated through two classical pathways: intrinsic (mitochondrial) pathway and/or extrinsic (death receptor) pathway [7]. In the intrinsic pathway, mitochondrial outer membrane permeabilization (MOMP) responds to multiple apoptotic stimuli, leading to the release of pro-apoptotic proteins including cytochrome c (Cyt c), which contributes to the formation of apoptosome composed of Cyt c, apoptotic protease-activating factor-1 (Apaf-1) and procaspase-9 [8,9]. The Bcl-2 family members have been considered as key regulators in MOMP change. They are generally grouped into 3 classes, anti-apoptosis class (BCL-2, BCL-XL, MCL-1, etc.), pro-apoptosis class (BAX, BAK), and BH3-only proteins (BAD, BIK, BID, BIM, BOK, etc.) which bind and regulate the anti-apoptotic BCL-2 proteins to promote apoptosis [10–12]. In the extrinsic pathway, the activation of cell surface death receptor such as Fas and tumor necrosis factor (TNF) receptor results in the formation of a death-inducing signaling complex (DISC) and the activation of caspase-8 [13]. Both of the two major pathways lead to the activation of caspase cascade and trigger the cell apoptosis in an irreversible way [14].

Arctigenin (ARG), a phenylpropanoid dibenzylbutyrolactone lignin, exists in a variety of traditional Chinese herbs including Bardanae fructus, Saussurea medusa, Arctium lappaL., T. nucifera, Forsythia intermedia and tropical climbing shrub Ipomea cairica [15–17]. Over the last decade, numerous experimental studies have demonstrated that ARG plays the roles of antioxidant, anti-inflammatory, antiviral and anti-tumor [17–21]. Of these, the anti-tumor activity of ARG attracts extensive attention. Previous studies showed that ARG exhibited a dramatically different cytotoxicity on different cancer cells. Yuan Gu et al. found out that ARG selectively promoted glucose-starved human adenocarcinoma cell A549 to undergo necrosis by inhibiting mitochondrial respiration [22]. Kim JY et al. reported that ARG could inhibit glucose deprivation-induced unfolded protein response [18] and two research groups revealed that ARG caused cell death through inducing cell cycle arrest or apoptosis in gastric cancer cells and lung adenocarcinoma cells [23,24]. Recently, Chia-Jung Hsieh et al. demonstrated that ARG activated the ROS/p38 MAPK pathway to induce apoptosis in human breast cancer MDA-MB-231 cells by triggering the mitochondrial caspase-independent apoptotic pathway both in vitro and in vivo [25]. All these works suggest that the mechanism of action of ARG is likely to vary among tissues and cancer cell types. However, to our knowledge, the anti-cancer potential of ARG has not been evaluated on HCC cells.

In this study, we used two HCC cell lines (Hep G2 and SMMC7721) to investigate the anti-hepatoma potential of ARG and the molecular mechanisms involved in this process. Our results demonstrated that ARG could induce apoptosis in both Hep G2 cells and SMMC7721 cells through activation of mitochondria- and Fas/ FasL-mediated pathways. Notably, ARG-induced apoptotic effect on Hep G2 was stronger than that on SMMC7721. That might because ARG exerted different effects on apoptosis-related factors, e.g., PI3K/p-Akt, NF-κB and p53, in Hep G2 and SMMC7721.

## Materials and Methods

### Reagents

RPMI-1640 medium, Dulbecco's modified Eagle's medium (DMEM) were obtained from Sigma-Aldrich (St. Louis, MO) and fetal bovine serum (FBS) were purchased from Gibco/BRL (Gaithersburg, MD, USA). Caspase-8, -9 Colorimetric Assay Kits and Mitochondria/Cytosol Fractionation Kit were purchased from BioVision (Milpitas, CA, USA). Rabbit monoclonal anti-Bcl-2, anti-Bax, anti-PI3K and rabbit polyclonal anti-cleaved caspase 3, anti-pro-caspase 3 were purchased from Cell Signaling Technology (Beverly, MA, USA). Mouse monoclonal anti-NF-κB, anti-Fas, anti-cleaved PARP-1, anti-β-actin and rabbit polyclonal anti-FasL were purchased from Santa Cruz Biotechnology (Santa Cruz, CA, USA). Rabbit monoclonal anti-p53, anti-pro caspase 9 and rabbit polyclonal anti-pro caspase 8 were purchased from Abcam (Cambridge, UK). Rabbit polyclonal anti-COX IV was purchased from ABclonal Biotech Co., Ltd (Beijing, China). Rabbit monoclonal anti-Akt, anti-p-Akt and anti-cytochrome C were purchased from Epitomics (Burlingame, CA, USA). Rabbit polyclonal anti-Histone 3 were purchased from Bioworld, Inc. (Louis Park, MN, USA). Dimethyl sulfoxide (DMSO), Hoechst33342,3-(4,5-dimethylthiazol-2-yl)-2,5-dephenyltetrazolium bromide (MTT), 5,5′,6,6′-tetrachloro-1,1′,3,3′-tetraethylbenzimidazolylcarbocyanine Iodide (JC-1) were obtained from Sigma–Aldrich (St. Louis, MO). All the other chemicals and reagents used in this experiment were of highest quality and obtained from standard commercial sources.

Arctigenin (ARG) (purity ≥ 98%) was purchased from JCKY Institute of Chemical Technology (Beijing, China). A 20 mM stock solution of ARG was prepared in DMSO and stored at -20°C. For all experiments, the desired concentrations of ARG were freshly diluted from the stock with DMEM before use.

### Cell culture

HCC cell lines Hep G2, SMMC7721 and normal human hepatic cell line LO2 were purchased from ATCC of China. Hep G2 and SMMC-7721 were cultured in DMEM supplemented with 12%- 15% heat-inactivated FBS (v/v), 100 U/ml penicillin and 100 μg/ml streptomycin. LO2 cells were cultured in RPMI 1640 medium supplemented with 10% heat-inactivated FBS (v/v), 100 U/ml penicillin and 100 μg/ml streptomycin. All the cells were incubated in a humidified incubator at 37°C with 5% CO_2_. Then, cells in the logarithmic growth phase were collected for the following experiments.

### Measurement of cell viability

Cell viability was determined using the MTT assay. Briefly, cells were seeded in a 96-well microplate at a density of 10^4^ cells/well and grew to 80% confluence. The cells were treated with different concentrations of ARG or vehicle for indicated durations. After treatment, cells were incubated with 1 mg/mL MTT in complete growth medium at 37°C for 4 h and then the culture medium was removed. Finally, 150 mL of DMSO was added to each well to dissolve the formazan crystals generated by live cells. Absorbance at the wavelength of 490 nm was read in an ELISA reader. Wells containing culture medium and MTT but no cells acted as blank and wells with cells which were cultured as normal without ARG or vehicle were used as control. The cell viability inhibition ratio was calculated by the following equation:

$$\text{Cell viability inhibition}\;\left(\%\right)=\left({\text{OD}}_{\text{control}}{-\text{OD}}_{\text{treated}}\right)/\left({\text{OD}}_{\text{control}}{-\text{OD}}_{\text{blank}}\right)\times 100\%$$

As a measure of cytotoxicity, the concentration of ARG inhibiting cell growth by 50% (IC_50_ value) was calculated from dose-response curves. All determinations were performed in quintuplicate.

### Immunofluorescence of cleaved caspase-3

Hep G2 and SMMC7721 cells were were seeded into 24-well plate and cultured for 12 h until 50% confluence. After incubation with 20 μM ARG or vehicle for 48 h, the cells were washed in PBS and fixed in 4% paraformaldehyde for 15 min. Then the cells were washed 3 times with ice-cold PBS and permeated using 0.5% Triton X-100 for 20 min. After that the cells were washed in washing buffer (1% BSA in 0.05% PBS-Tween) and blocked for 30 min with 3% goat serum. Then the cells were incubated with a 1/100 dilution of cleaved caspase-3-specific antibody overnight at 4°C. The antibody was removed, and the cells were washed 3 times with PBS-Tween. A 1/100 dilution of anti-rabbit Cy3 was then incubated with the cells for 1 h at room temperature. The secondary antibody was removed, and the cells were washed 3 times with PBS-Tween. Then the cells were incubated with Hoechst 33342 (5 μg/mL) at room temperature for 20 min in the dark. After being washed with PBS, the cellular fluorescent changes were observed under a fluorescence microscope.

### Flow cytometric analysis of apoptosis

Hep G2, SMMC7721 and LO2 cells were incubated with ARG at different concentrations for 24 h. Apoptotic cells were identified by an annexin V and PI staining kit (BioVision) according to manufacturer’s instructions. Briefly, after treatment, cells were trypsinized and washed twice with ice-cold PBS. Then the washed cells were gently resuspended in annexin V-binding buffer, and incubated with annexinV-FITC and PI in dark for 10 min. Finally, the stained cells were measured by flow cytometry (Becton Dickinson, San Jose, CA). Annexin V+/PI- cells were considered as an early stage of apoptosis and Annexin V+/PI+ cells were considered as late apoptotic cells.

### Mitochondrial membrane potential (Δ*Ψ*_*m*_) assay

Mitochondrial membrane potential disruption during apoptosis was estimated using the fluorescent cationic dye JC-1. Hep G2 and SMMC7721 cells were seeded in a 6-well plate for 24 h, then exposed to different concentrations of ARG for 12 h and 24 h respectively at 37°C. After treatment, cells were harvested and washed twice with cold PBS, then re-suspended in JC-1 reagent solution and incubated at 37°C in dark for 20 min. Finally, cells were washed and re-suspended in PBS and analyzed by flow cytometry.

### Caspase activity analysis

Caspase-8 and caspase-9 Colorimetric Assay Kits (BioVision) were used to evaluate caspase-8 and -9 activities, respectively. After ARG treatment, the cell lysates were prepared following manufacturer's instructions. Then, the release of ρ-nitroaniline (ρNA) was measured at 405 nm with a spectrophotometer. Results are represented as the percentage of change in activity compared with the untreated control.

### Protein extraction and Western blot analysis

Cells were collected and washed twice in ice-cold PBS, then incubated in lysis buffer on ice for 1 h. Then cell lysates were centrifuged at 12,000 rpm for 15 min at 4°C. For NF-κB, both cytoplasmic and nuclear extracts were collected separately using nuclear and cytoplasmic extraction kit (Thermo Scientific). For Cyt C analysis, both cytoplasmic and mitochondrial extracts were collected separately using Mitochondria/Cytosol Fractionation Kit (BioVision, USA). Protein concentration was measured by the Bradford assay. Equal amounts of proteins were separated using SDS-PAGE and electrophoretically transferred to PVDF membrane (Millipore). The membranes were blocked with 5% non-fat milk in TBST for 1 h at room temperature, and then incubated with the appropriate primary antibody at 4°C overnight. After being washed, the blots were incubated with peroxidase-conjugated secondary antibody at 37°C for 1 h. Bands were monitored using Western blot ECL reagent (Thermo Scientific). Densitometric analysis was carried out using ChemiDoC XRS+ system (BioRad, Laboratories, Hercules, CA).

### Statistical analysis

All experiments were performed in triplicate and the results were expressed as mean ± SD. Statistical analysis was performed by ANOVA followed by Dunnett’s test using SPSS.15.0 software. P< 0.05 was considered statistically significant.

## Results

### Cytotoxic effect of ARG on HCC cells Hep G2, SMMC7721 and normal hepatic cell line LO2

**Fig 1:**
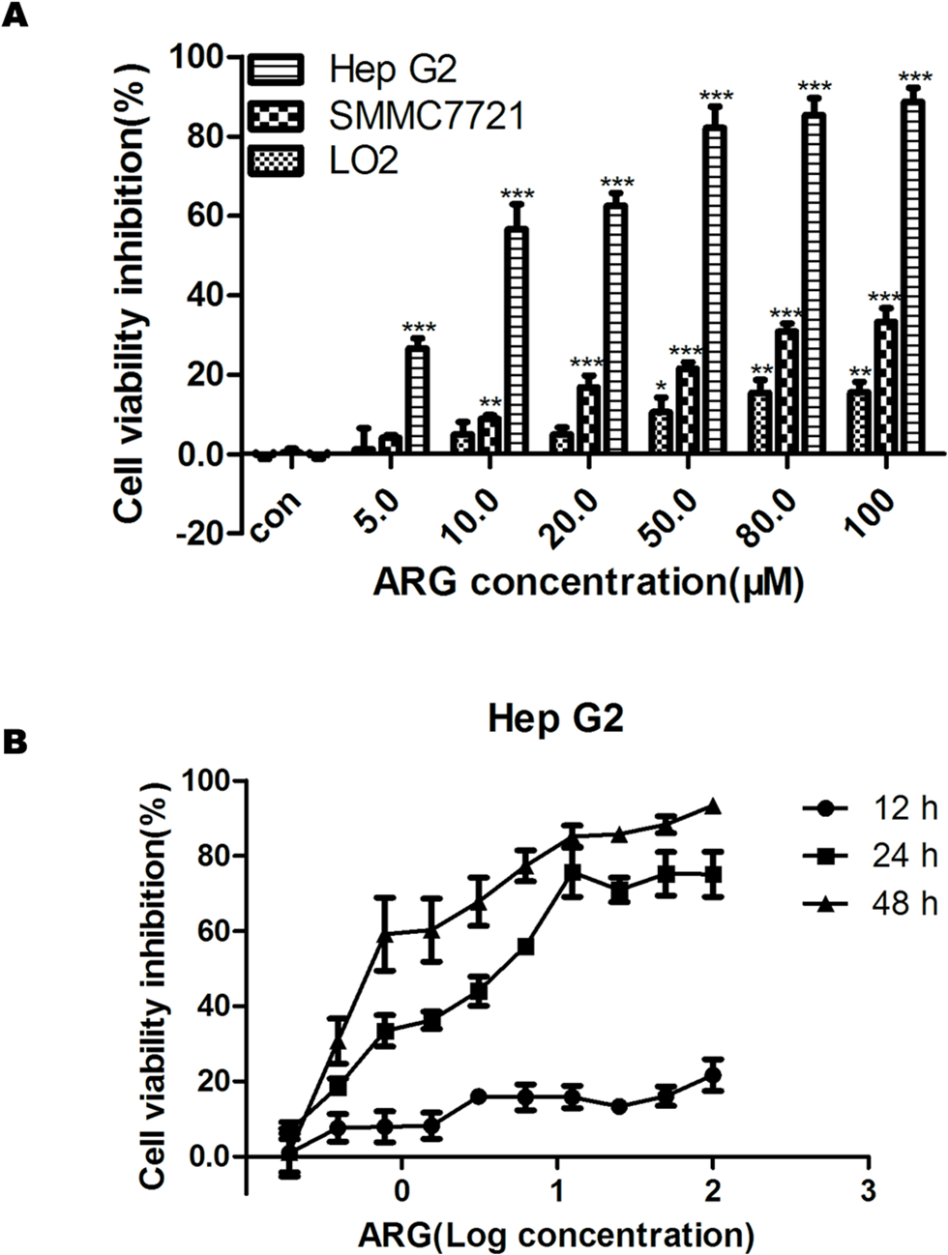
Cytotoxic effect of ARG on HCC cells and normal hepatic cells. (A) Hep G2, SMMC7721 and LO2 cell lines were treated with ARG at 5, 10, 20, 50, 80, 100 μM for 24 h. (B) Hep G2 cells were exposed to a gradient dose of ARG for the indicated time period and IC_50_ value was calculated for each time point. Cell viability inhibition was assessed by MTT assay. Each value is the mean ± SD of five independent experiments. *p<0.05, **p<0.01, ***p<0.0001 significant difference between control and ARG-treated cells in each cell line, as analyzed by Dunnett’s Multiple Comparion Test.

Three cell lines including HCC cells Hep G2 and SMMC7721, and normal hepatic cell LO2 were chosen for investigating cytotoxic effect of ARG. As shown in Fig 1A, after treatment of ARG at the concentrations of 5, 10, 20, 50, 80, 100 μM for 24 h, the inhibitory rates were respectively 26.61%, 56.60%, 62.53%, 82.11%, 85.38% and 88.69% in Hep G2 cells. However, SMMC7721, another hepatoma carcinoma cell line, was less sensitive to ARG compared with Hep G2. For instance, the cell viability was only reduced by 8.83% in 10 μM, 16.81% in 20 μM, 21.56% in 50 μM, 30.87% in 80 μM and 33.28% in 100 μM ARG medium. No difference was observed between the control group and 5 μM ARG-treated SMMC7721 group. For normal hepatic cells, cell cytotoxic effect was only found in groups treated with 50 μM, 80 μM and 100 μM ARG. Fig 1B shows that ARG could inhibit the growth of Hep G2 cells not only in a dose-dependent manner, but also in a time-dependent. The IC_50_ values after 12 h, 24 h and 48 h of ARG treatment were respectively 38.29 μM, 1.99 μM and 0.24 μM.

### ARG induced apoptotic cell death in HCC cells

**Fig 2:**
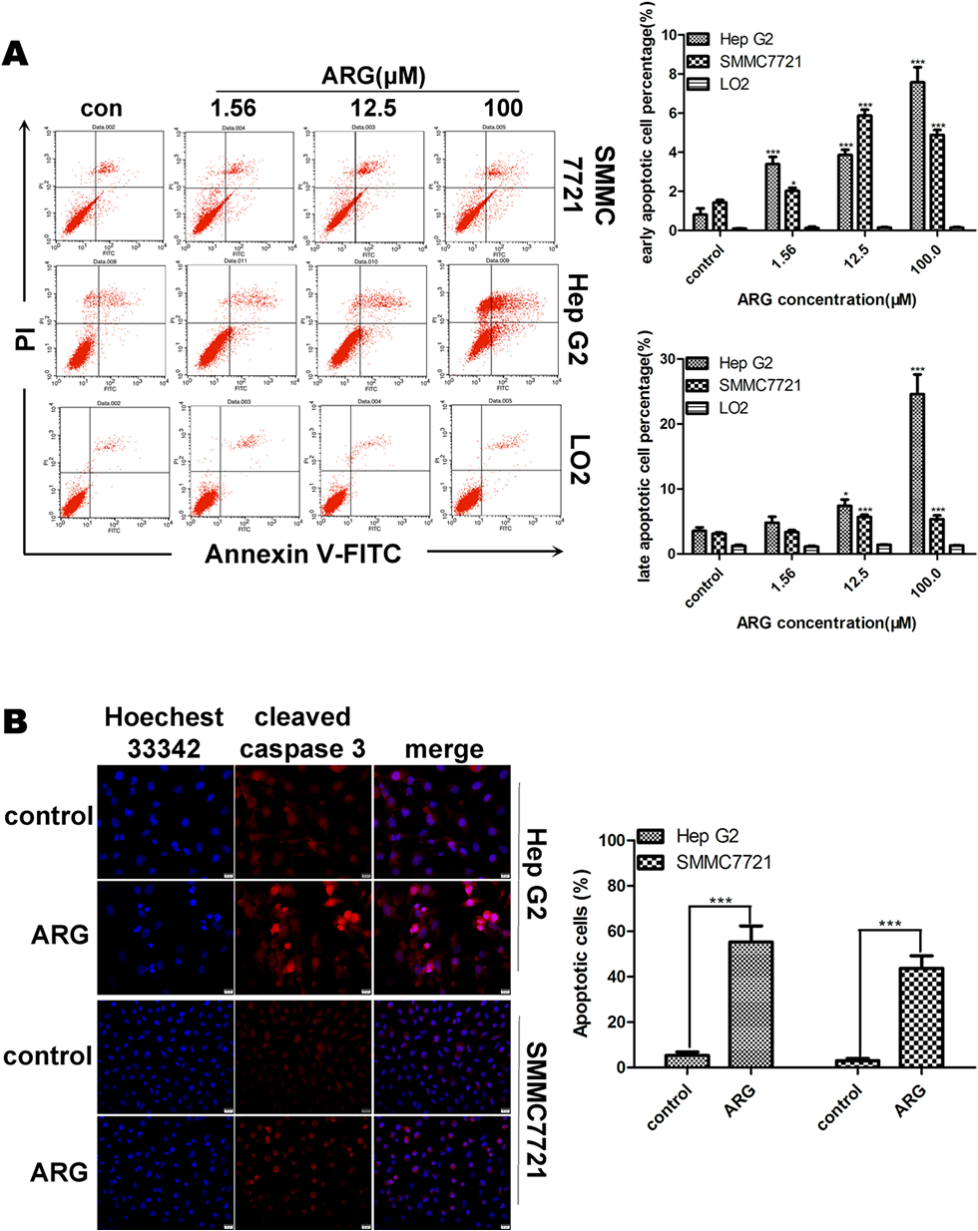
ARG promotes HCC cells to undergo apoptosis. (A) Hep G2, SMMC7721 and LO2 cells treated with ARG (0, 1.56, 12.5, 100 μM) for 24 h were double-stained with Annexin V-FITC/PI and analyzed by flow cytometry. Quantification of population rate of early apoptotic cells (Annexin V+/PI− cells, lower right quadrant) and late apoptotic cells (Annexin V+/PI+ cells, upper right quadrants) were shown. Data represent the mean ± SD of triplicate experiments. Significant differences from vehicle-treated control were indicated as *p<0.05, **P<0.01, ***P<0.0001 in each cell line. (B) HepG2 and SMMC7721 cells were treated with ARG at the concentration of 20 μM for 48 h. The nuclei morphology changes were analyzed by Hoechst 33342 staining and the immunofluorescence analysis of cleaved caspase-3 are visualized using the cleaved caspase-3 antibody. The cellular fluorescent changes were observed by fluorescence microscope. At least 200 cells were counted to score the percentage of apoptotic cells which contained both cleaved caspase-3 and condensed and highly fluorescent nuclei fragments in each treatment group. Significant differences from control group were indicated as ***P<0.0001 in each cell line.

To characterize the mechanism involved in ARG-induced cells death, we assessed if the effect of ARG on Hep G2, SMMC7721 and LO2 was due to apoptosis. Cells were double stained for Annexin V-FITC/PI and analysed by flow cytometry after the exposure of ARG (0, 1.56, 12.5, 100 μM) for 24 h. As shown in Fig 2A, ARG induced apoptosis in HCC cells (Hep G2, SMMC7721) in dose-dependent manner but not in LO2 cells. For both Hep G2 and SMMC7721 cells, 12.5 and 100 μM ARG treated groups showed a significant difference in terms of quantity of late apoptotic cells compared with the control groups. While the percentages of early apoptotic cells in the three ARG treated groups were all significantly higher than those of the control groups. However, it was noted that 1.56–100 μM ARG did not cause any significant apoptosis in normal human hepatic cells LO2.

Both nuclei fragmentation with condensed chromatin and the cleavage of caspase 3 are well-known characteristic of apoptosis. Fluorescence staining was performed to detect the abnormalities of nuclear morphology and expression of cleaved caspase 3 induced by ARG. As shown in Fig 2B, treatment of Hep G2 and SMMC7721 cells with ARG resulted in a significantly increase in the number of apoptotic cells. Some cells displayed a typical apoptotic morphological changes (condensed and highly fluorescent nuclei fragments) and contained cleaved caspase-3. These data confirmed ARG induced apoptotic cell death in HCC cells.

### ARG trigger apoptosis via a mitochondrial dependent pathway in HCC cells

**Fig 3:**
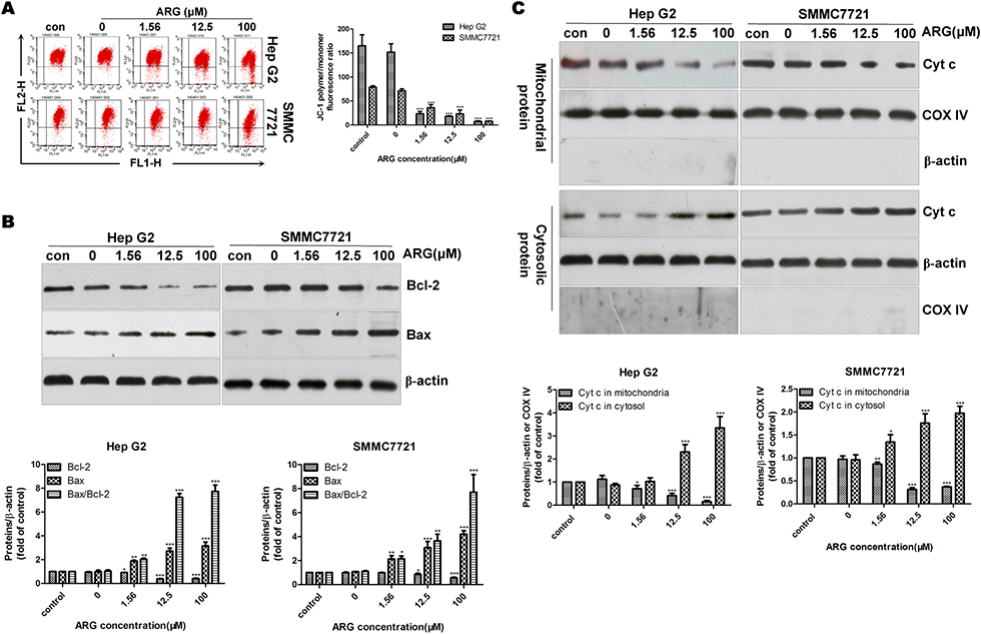
ARG caused the disfunction of mitochondrial membrane in HCC cells. Hep G2 and SMMC7721 cells were exposed to ARG (0, 1.56, 12.5, 100 μM) for 12 h and 24 h respectively. (A) Flow cytometric detection of Δ*Ψ*_*m*_ in HCC cells using JC-1. (B) The expression of Bcl-2 and Bax were analyzed by western blot with the loading control of β-actin. (C) Mitochondrial and cytoplasmic extracts were measured for Cyt c levels. Purity of the extracts was verified by β-actin (cytoplasmic) and COX IV (mitochondrial) expressions. Data were represented as mean ± SD of three separate experiments. Significant differences from control were indicated *p<0.05, **p<0.01, ***p<0.0001.

It has been generally agreed that mitochondrial dysfunction participated in the induction of apoptosis and even played a pivotal role in the apoptotic process [26–28]. To determine whether ARG induces apoptosis by triggering the mitochondrial apoptosis pathway, we have investigated decrease of mitochondrial transmembrane potential (Δ*Ψ*_m_), the changes in the expression of the Bcl-2 family proteins and the release of Cyt c. JC-1 was used to detect the loss of mitochondrial transmembrane potential (Δ*Ψ*_m_) in HCC cells treated with different concentrations of ARG. Data in Fig 3A revealed that ARG caused an obvious decrease of Δ*Ψ*_m_ compared with the corresponding control group in both Hep G2 and SMMC7721 cells in a dose-dependent manner. Breaking the balance between pro-apoptotic Bax and anti-apoptotic Bcl-2 proteins induces mitochondrial permeabilization, which results in the release of a plethora of proapoptotic factors from the intermembrane space and subsequent activation of apoptosis [29]. Western blot analysis in Fig 3B showed that treatment of Hep G2 cells and SMMC7721 cells with ARG increased Bax protein levels after 12 h- and 24 h-exposure respectively and in contrast, ARG markedly decreased Bcl-2 expression, which led to an increase in the Bax/Bcl-2 ratio in a dose-dependent manner. In addition, in ARG treated HCC cells, more Cyt c was released from mitochondria to cytosol with increasing ARG concentration as shown in Fig 3C. All the data above indicated that ARG trigger apoptosis via a mitochondrial dependent pathway in HCC cells.

### ARG also provokes apoptosis in HCC cells through Fas/FasL-mediated pathway

**Fig 4:**
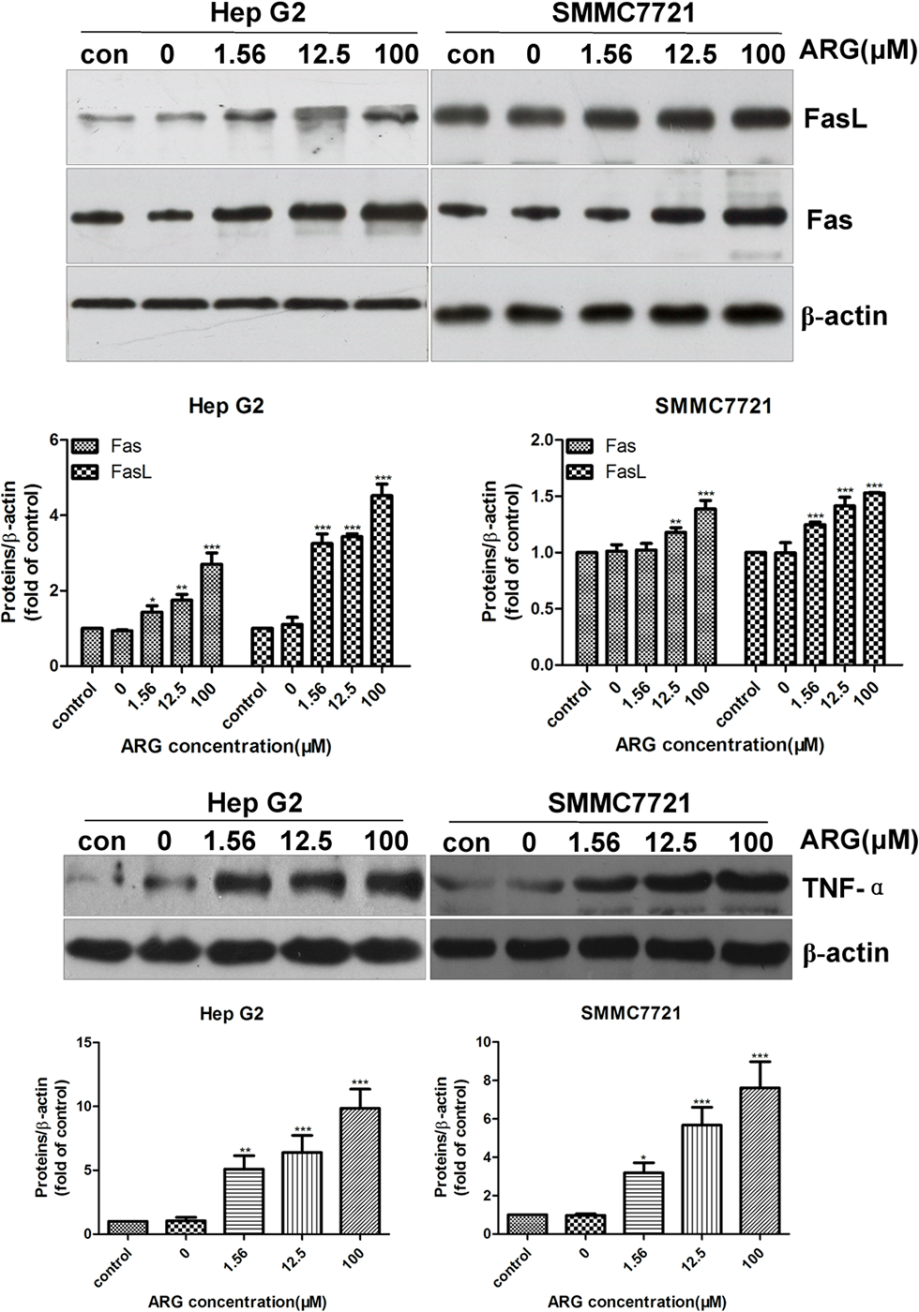
ARG induced the changes of apoptotic proteins related to extrinsic pathways. Hep G2 and SMMC7721 cells were incubated with ARG (0, 1.56, 12.5, 100 μM) for 12 h and 24 h respectively, and then harvested for investigating the TNF-α, Fas and FasL expression level by western blot. The data represent mean ± SD of three separate experiments. Significant differences from control were indicated as *p<0.05, **p<0.01, ***p<0.0001.

Many researches pointed out that apoptosis was induced via two main routes, mitochondria-mediated endogenous and Fas/FasL-mediated exogenous ones. Thus, we explored the possibility that ARG could induce apoptosis through the extrinsic pathway. Hep G2 and SMMC7721 cells were exposed to various concentrations (0, 1.56, 12.5 and 100 μM) of ARG for 12 h and 24 h respectively in order to examine the extrinsic molecular regulators. Our data showed that ARG dramatically stimulated the expression of TNF-α, Fas/FasL in both Hep G2 cells and SMMC7721 cells in a dose-dependent manner shown in Fig 4. Thus, we could speculate that ARG was also able to induce HCC cells to undergo apoptosis via Fas/FasL-mediated pathway.

### The cleavage of poly (ADP-ribose) polymerase and the activation of caspases

**Fig 5:**
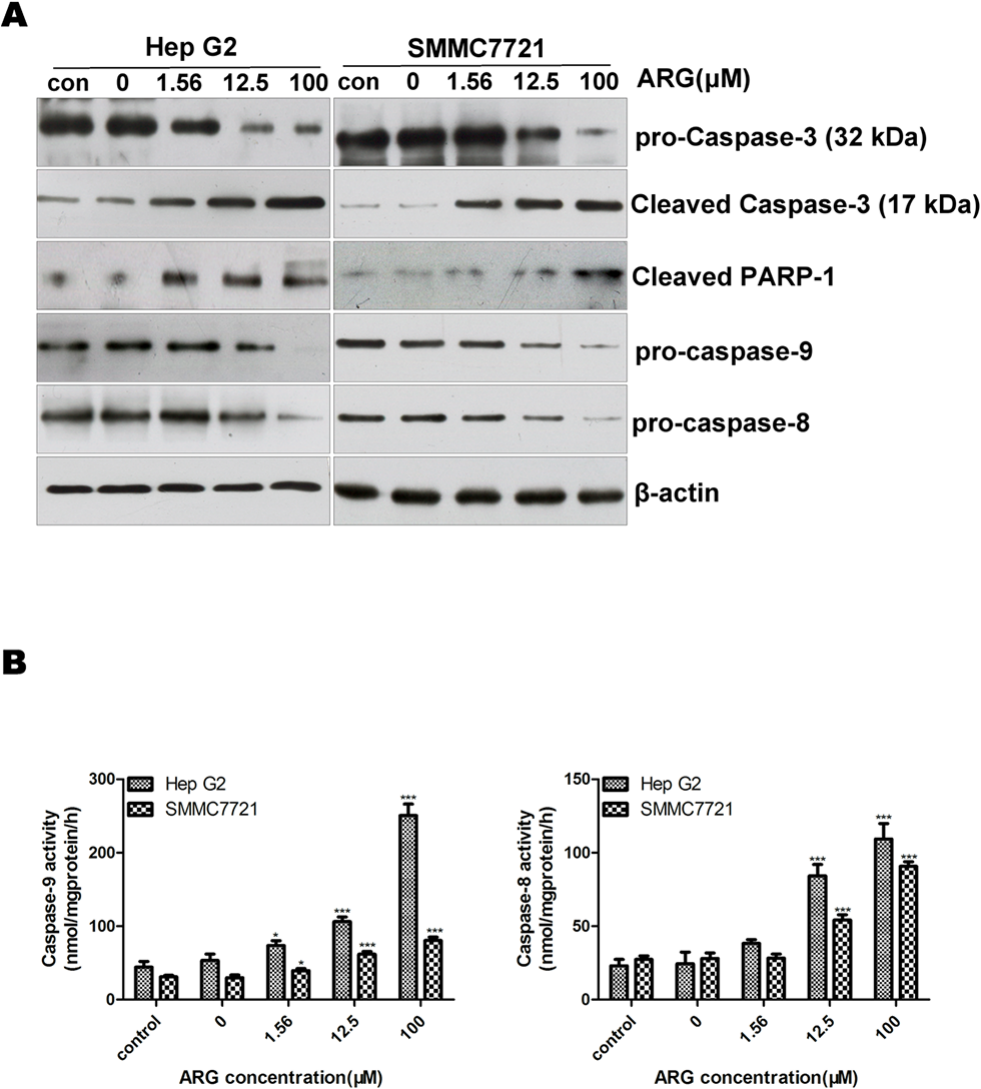
Effect of ARG on the activation of caspases. After Hep G2 and SMMC7721 cells were treated with ARG (0, 1.56, 12.5, 100 μM) for 12 h and 24 h respectively. The protein levels of caspase-3, cleaved PARP-1, pro-caspase -8, -9 were analyzed by western blot analysis (A) and the activities of caspase-8, -9 were detected by Caspase Colorimetric Assay Kits (B). The data represent mean ± SD (n = 3). *p< 0.05, **p< 0.01, ***p< 0.0001 vs. untreated control.

Caspase activation and PARP-1 cleavage are known as a major step in apoptosis. We investigated the expression levels of pro-caspase-9, pro-caspase-8, caspase-3 and cleaved PARP-1 in HCC cells treated with ARG. Meanwhile the enzymatic activities of caspase-8 and -9 were measured by Caspase Colorimetric Assay Kits. As shown in Fig 5A, ARG treatment significantly induced a decrease in the protein levels of pro-caspase-8, -9 in a dose-dependent manner with the increasing enzymatic activities of caspase-8, -9 (Fig 5B). Furthermore, ARG treatment could lead to a processing of caspase-3 to its cleaved form and the cleavage of PARP-1. These results further confirmed that ARG-induced apoptosis might proceed by both intrinsic and extrinsic pathways.

### NF-κB, p53 and PI3K/Akt pathways are invovled in the Hep G2 apoptosis induced by ARG

**Fig 6:**
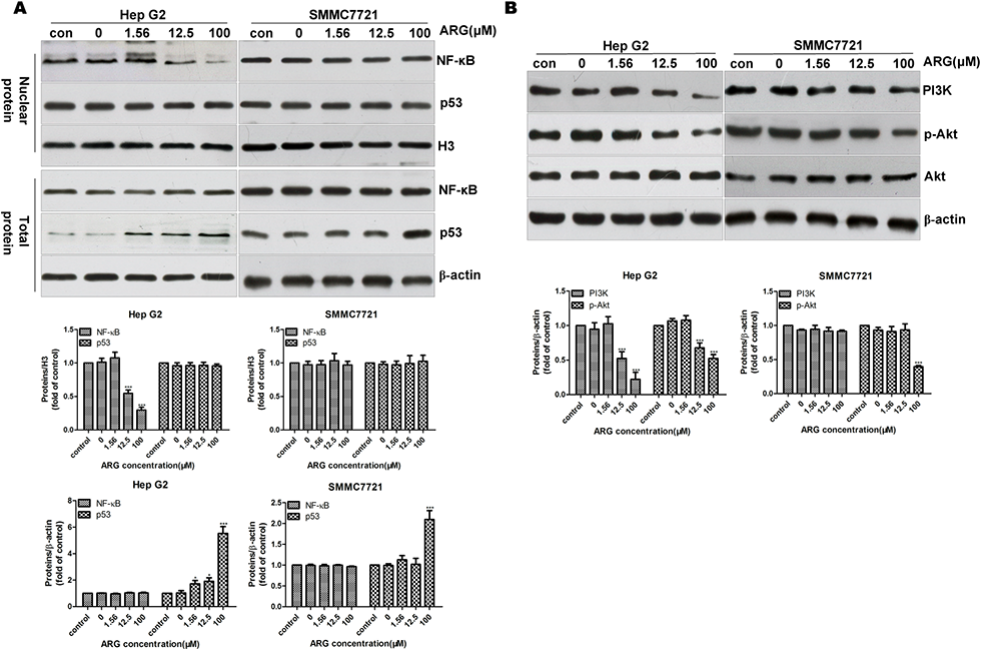
Effect of ARG on NF-κB, p53 and PI3K/AKT pathways in HCC cells. Hep G2 and SMMC7721 cells were exposed to ARG (0, 1.56, 12.5, 100 μM) for 12 h and 24 h respectively. Western blot analysis was performed. (A) NF-κB (p65) and p53 in nuclear, total NF-κB (p65) and p53 expression levels were shown. (B) PI3K, Akt and p-Akt expression levels were shown. Histone 3 was used as a loading control for nuclear protein, and β-actin for whole cell protein. The data shown are the representative image from three independent experiments. *p<0.05, **p<0.01, ***p<0.0001 significant differences from control.

As shown in Fig 6A, ARG treatment led to a dose-dependent down-regulated nucleus translocation of NF-κB in Hep G2 cells, which was consistent with the results of several reported researches on other anticancer drugs [30,31]. Interestingly, no significant difference was found in SMMC7721 cells. The total amount of NF-κB in whole cell lysate remained unchanged in both of the two cell lines. As we know, p53 functions as a central mediator for organizing cell responses to various stress and anticancer drugs that lead to apoptosis, G1-phase arrest, and DNA repair [32]. It also regulates the expression of many genes whose products are involved in apoptosis [33–35]. Our data in Fig 6A demonstrated that ARG treatment resulted in an accumulation of p53 in total protein in a dose-dependent manner especially in Hep G2 cells. However, we did not find any significant changes in nuclear.

The PI3K/Akt signaling pathway is a well-known survival pathway which is associated with protection against apoptosis, regulation of cell cycle, cellular transformation, cell growth, and tumorigenesis [49–51]. To analyze whether the inhibition of PI3K and Ser473 phosphorylation of Akt was involved in ARG induced apoptosis, we measured the expression levels of PI3K, total and phosphorylated Akt by western blot. As shown in Fig 6B, ARG led to a dose-dependent suppression of phosphorylated Akt and PI3K in Hep G2 cells. But in SMMC7721 cells, the suppression effect could only be found in p-Akt at 100 μM dose. It suggested that ARG might induce apoptosis via deactivation of the PI3K/Akt pathway.

## Discussion

ARG, a phenylpropanoid dibenzylbutyrolactone lignan, is an active molecule isolated from dried fruit of *Arctium lappa L*. and has been previously reported to exert high biological activities including antioxidant, anti-inflammatory, antiviral and anti-tumor activities [17–21]. It can selectively inhibit the growth in multiple carcinoma cells while sparing normal cells [23,25]. Thus ARG has greatly spurred our attention as a promising anti-neoplastic drug. In our study, we analysed the cytotoxicity of ARG on Hep G2, SMMC7721 and normal human hepatic cells (LO2) *in vitro*. Our results clearly demonstrated that ARG markedly inhibited the growth of Hep G2 and SMMC7721 cells, but no inhibitory effect was observed on normal human hepatic cells (LO2) until the concentration of ARG was beyond 50 μM. This demonstrated that ARG possessed selective cytotoxicity between normal and cancer cells within a certain concentration range. Additionally, ARG did not show a growth inhibitory effect on SMMC7721 as strong as on Hep G2 cells. Therefore, we know that the ARG-sensitivities of different HCC cells differ, and the difference may come from the difference of their own.

There are several different types of cell death reported in current studies, such as apoptosis, autophagy and necrosis [36]. As we know, cell apoptosis is a main approach in the clinical therapies of various carcinoma [37]. Our results demonstrated that ARG induced apoptosis in HCC cells, but hardly in normal human hepatic cells. This is in a good agreement with our expectation that cancer cells are killed by ARG while normal cells are less affected by the treatment.

Targeting apoptotic pathways in premalignant and malignant cells is an effective strategy for cancer prevention and treatment [38]. Our study focused on the mechanism of apoptosis induced by ARG in HCC cells. The regulation of intracellular apoptosis process is complex and multifaceted. Our study is the first to prove that both intrinsic and extrinsic pathways might be involved in ARG-induced HCC cells apoptosis. In the intrinsic apoptotic pathway, mitochondria which are the center of energy and metabolism of eukaryotic cells, also play a critical role in processing the apoptosis signal [39]. The loss of Δ*Ψ*_m_ and the disruption of MOMP are the central gate in turning on/off apoptosis and recognized as the key step in the apoptotic cascade activation [40]. We firstly detected Δ*Ψ*_m_ in HCC cells treated with ARG. As expected, ARG treatment resulted in a dose-dependent loss of Δ*Ψ*_m_ (Fig 3A). It has been reported that Bcl-2 family, which was composed of both pro-apoptotic molecules (Bax, Bcl-Xs, Bak, Bid, Bad, Bim, Bik) and anti-apoptotic molecules (Bcl-2, Bcl-XL, Bcl-W, Mcl-1, A1), controlled the release of mitochondrial cytochrome c by modulating the permeability of the outer mitochondrial membrane [41]. Bax and Bcl-2 are key components of apoptosis mediated by the mitochondria. In the present study, the disruption of MOMP was determined by the decrease in Bcl-2 level and the increase in Bax level which led to an unbalance between the pro-apoptotic molecules and anti-apoptotic molecules (Fig 3B). Once mitochondrial membrane function brake down, Cyt c is released from the mitochondria into the cytosol (Fig 3C). Then the apoptosome is formed through the binding of Cyt c and Apaf-1. The apoptosome recruits multiple pro-caspase-9 molecules and promotes their cleavage to an active form, known as the initiators of apoptosis [42].

Different from intrinsic apoptotic pathway, the extrinsic apoptotic pathway is initiated by Fas (CD95 or APO-1), which is a cell surface receptor belonging to the TNF receptor superfamily [43]. Fas is activated by ligation with Fas ligand, which results in the aggregation of its intracellular death domains, leading to the formation of DISC [13]. Then the DISC recruits pro-caspase-8 and activates caspase-8, then caspase-3 [44]. There, increasing evidence demonstrated that TNF-α induced cell death through binding with CD120a leading to an activation of caspase cascade [45]. We found upregulated expression of TNF-α, Fas and FasL (Fig 4), reflecting the participation of the extrinsic pathway, which was further confirmed by activation of caspase-8 (Fig 5B). All the above investigations indicated that the apoptosis of HCC cells induced by ARG was through both intrinsic and extrinsic pathways.

In addition to the pro-apoptotic pathways, some survival signals such as PI3K/Akt pathway, which is becoming a target for treating various tumors [46], are also involved in the apoptosis caused by ARG in HCC cells. PI3K is a dimeric enzyme composed of an inhibitory/regulatory (p85) and a catalytic (p110) subunit. The p85 subunit is anchored to erbB receptor docking sites, and the p110 subunit is responsible for the phosphorylation and activation of the protein serine/threonine kinase Akt which is the central downstream effector molecule of the PI3K pathway [47]. In many cancer cells, activated Akt phosphorylates various substrates either in the cytoplasm or in the nucleus, and it inhibits apoptosis through multiple pathways [48] including inducing the direct phosphorylation and inactivation of many pro-apoptotic proteins like Bad and caspase-9 [49], down-regulation of some pro-apoptotic proteins and the up-regulation of some anti-apoptotic proteins, such as Bcl-2 [50,51]. In our study, Akt inactivation was only detected in HCC cells (Fig 6B). But in LO2 cells, ARG treatment resulted in a dose-dependent activation of Akt (S1 Fig). These findings might help us explain the reason that ARG preferentially induce cell apoptosis in HCC cells. It is worth noting that PI3K was not found decreased in SMMC7721 as in Hep G2 under ARG treatment (Fig 6B), and PI3K inhibitor LY294002 could only enhance the inhibitory effect of ARG on Hep G2 not in SMMC7721 (S2 Fig). That might be related to the difference in susceptibility of HCC cells.

Literature reports suggest that p53 plays a key role in the regulation of apoptosis via decreasing the expression of the apoptosis-suppressing gene Bcl-2, meanwhile increasing the expression of Bax that encodes a dominant inhibitor of the Bcl-2 protein [52]. In our study, over-expression of p53 in whole cell protein without any significant change in nuclear (Fig 6A) was found after ARG treatment. Based on our results obtained in mitochondrial apoptosis pathway, we can presume that the role of up-regulated p53 is consistent with the previous observations of a crucial role of p53 in mediating cell apoptosis via inhibition and interaction with Bcl-2 or activating Bax in mitochondria [53–55]. NF-κB is also known for its anti-apoptotic function of transcriptional regulating of various anti-apoptotic genes involved in survival signaling [56,57], and NF-κB-linked pathway was reported to be involved in various drugs induced apoptosis [58–61]. Previous study on HCC cells indicates that constitutive NF-κB activity plays an anti-apoptotic role [62]. However, Li et al., [63] proved that the flavonoid chrysin sensitizes several human cancer lines, including Hep G2, to apoptosis via suppression of NF-κB. Here our data demonstrated that ARG possessed an inhibitory effect on NF-κB activation in Hep G2 but not in SMMC7721 cells, which might lead to different susceptibilities in HCC cells. Our data showed that ARG could induce increase in expression levels of pro-apoptotic factors like p53, Bax, Fas/FasL and caspase cascade with a concomitant decrease in levels of anti-apoptotic factor Bcl-2, p-Akt and NF-κB, which revealed a novel function of ARG and enhanced the value of ARG as a useful anti-cancer agent.

## Conclusion

**Fig 7:**
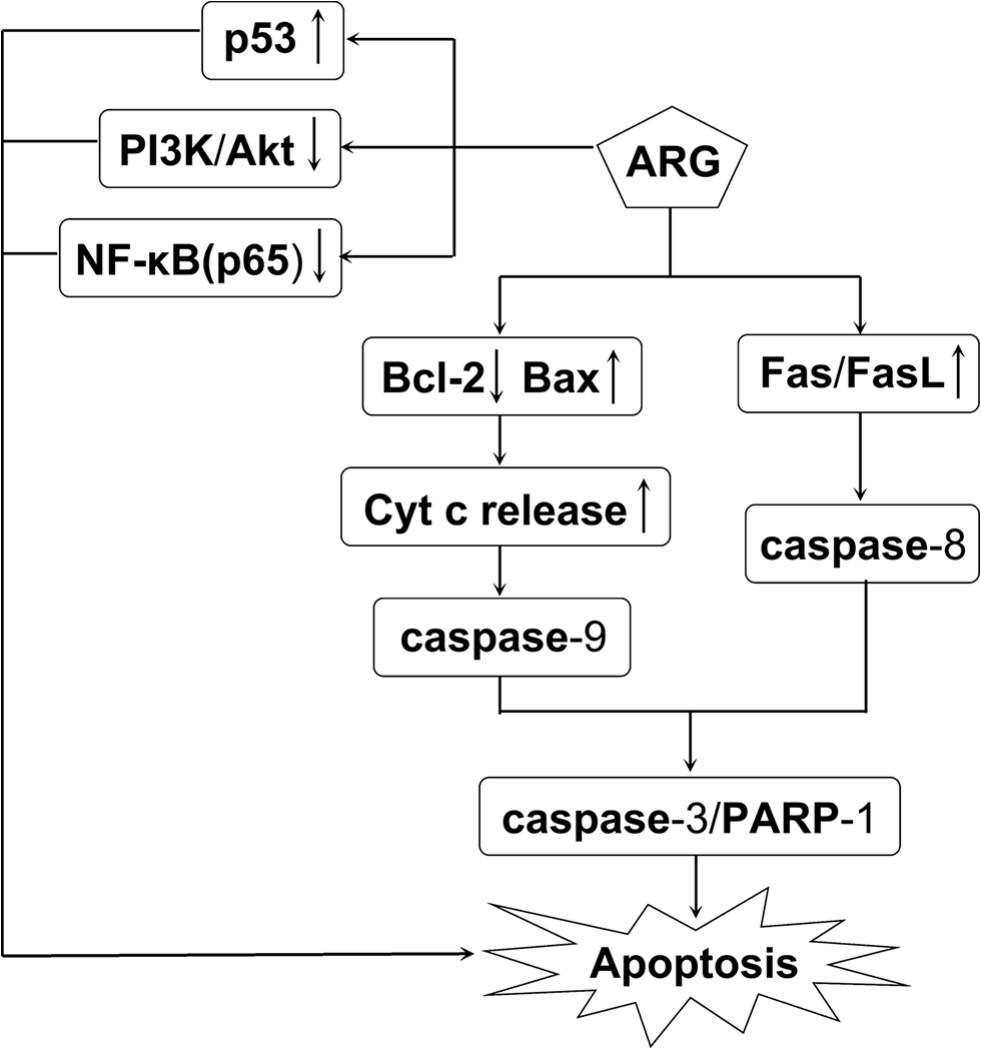
The apoptotic pathways induced by ARG in Hep G2 cell line.

In conclusion, our results demonstrated that ARG displayed cytotoxic activity against human HCC cells (Hep G2, SMMC7721). Our further study revealed that ARG might induce apoptosis in HCC cells via mitochondria and Fas/FasL-dependent apoptotic pathways. Inhibition of PI3K/Akt pathway, inactivation of NF-κB and activation of p53 may augment the apoptotic sensitivity of Hep G2 cells. These findings also defined the possible molecular mechanisms of ARG-induced cell death (Fig 7). Further studies are needed to examine the efficacy of ARG *in vivo*.

## Supporting Information

S1 Fig Effect of ARG PI3K/AKT pathways in LO2 cells. LO2 cells were exposed to ARG (0, 1.56, 12.5, 100 μM) for 24 h. Western blot analysis was performed. Akt and p-Akt expression levels were shown with a loading control of β-actin. The data shown are the representative image from three independent experiments. *p<0.05, **p<0.01, ***p<0.0001 significant differences from control.
(TIF)

S2 Fig The effect of ARG in combination with PI3K inhibitor on the proliferation of HCC cells. Hep G2, SMMC7721 cell lines were exposed to 20 μM ARG with or without PI3K inhibitor LY294002 for 24 h. Cell viability inhibition was assessed by MTT assay. Each value is the mean ± SD of five independent experiments. *p<0.05, **p<0.01, ***p<0.0001 significant difference between ARG combined with LY294002 group and ARG group in each cell line, as analyzed by Dunnett’s Multiple Comparion Test.
(TIF)
