# Supplementary figures and images for: Mechanism of Arctigenin-Induced Specific Cytotoxicity against Human Hepatocellular Carcinoma Cell Lines: Hep G2 and SMMC7721
Source: PLoS One. 2015 May 1;10(5):e0125727. doi: 10.1371/journal.pone.0125727 (PMC4416797; doi:10.1371/journal.pone.0125727)

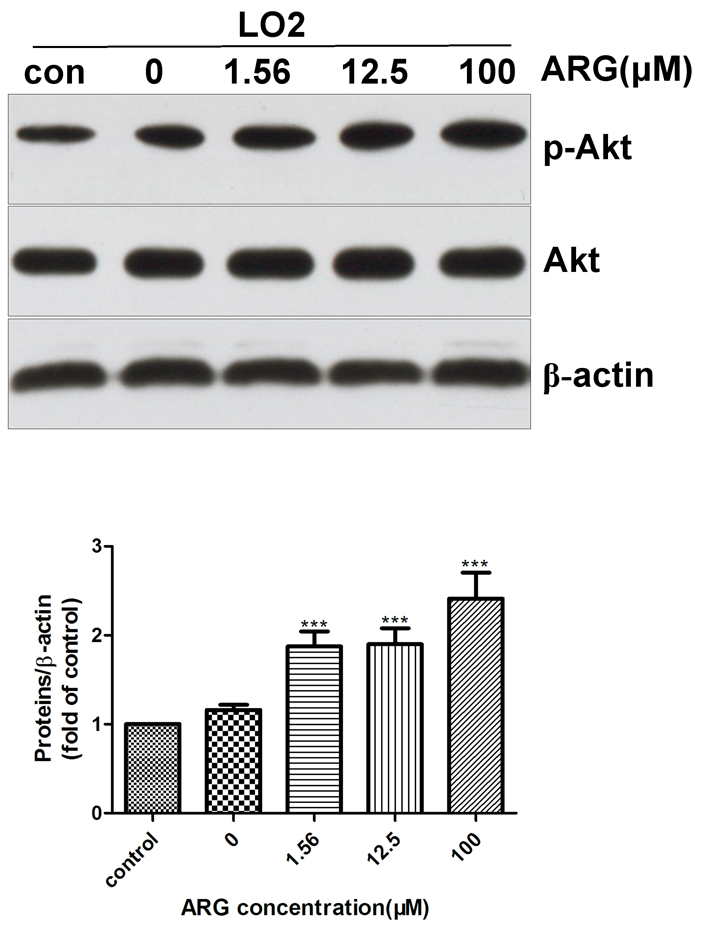

Supplement: S1 Fig — LO2 cells were exposed to ARG (0, 1.56, 12.5, 100 μM) for 24 h. Western blot analysis was performed. Akt and p-Akt expression levels were shown with a loading control of β-actin. The data shown are the representative image from three independent experiments. *p<0.05, **p<0.01, ***p<0.0001 significant differences from control. (TIF) [file pone.0125727.s001.tif]

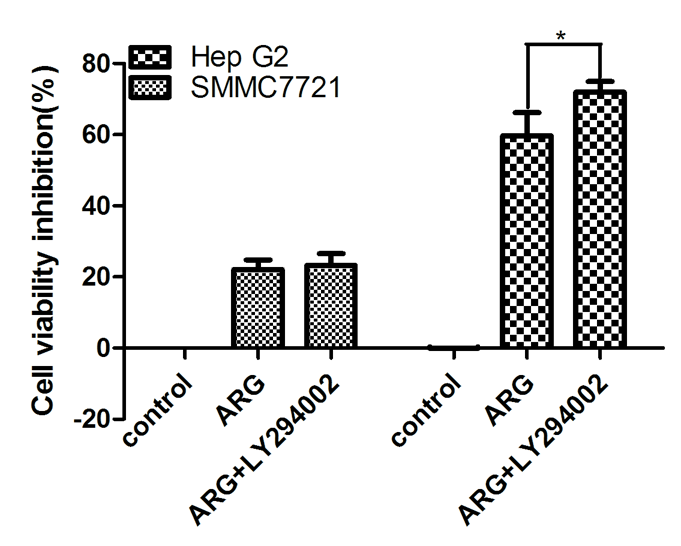

Supplement: S2 Fig — Hep G2, SMMC7721 cell lines were exposed to 20 μM ARG with or without PI3K inhibitor LY294002 for 24 h. Cell viability inhibition was assessed by MTT assay. Each value is the mean ± SD of five independent experiments. *p<0.05, **p<0.01, ***p<0.0001 significant difference between ARG combined with LY294002 group and ARG group in each cell line, as analyzed by Dunnett’s Multiple Comparion Test. (TIF) [file pone.0125727.s002.tif]
